# Best evidence summary of non-pharmacological interventions for postoperative abdominal distension in gynecological laparoscopic

**DOI:** 10.3389/fmed.2026.1863641

**Published:** 2026-08-18

**Authors:** Yidan Cao, Zhengqing Wang, Liping A, Xuefang Zhou, Jie Zhang

**Affiliations:** 1 School of Nursing, Ningxia Medical University, Yinchuan, China; 2 Department of Gynecology, Yinchuan First People’s Hospital, Second Clinical Medical College, Ningxia Medical University, Yinchuan, China

**Keywords:** abdominal distension, evidence summary, gynecology, laparoscopy, non-pharmacological interventions

## Abstract

**Objective:**

To retrieve and summarize the best evidence on non-pharmacological interventions for postoperative bloating in gynecological laparoscopic patients, providing an evidence-based foundation for the clinical management of gastrointestinal function.

**Methods:**

According to the 5S pyramid model of evidence-based practice resources, a systematic search was conducted in databases such as Web of Science, Embase, Cochrane Library, PubMed, CNKI, Wanfang, and VIP. Relevant guidelines, clinical decision, evidence summaries, systematic reviews, and expert consensus were collected. The search period spanned from the inception of the databases to January 31, 2026. Two reviewers independently screened and evaluated the literature, followed by evidence extraction and summarization according to the JBI Evidence Grading and Recommendation System.

**Result:**

A total of 20 articles were included, comprising 4 guidelines, 1 clinical decision, 5 expert consensus, 1 evidence summary, and 9 systematic reviews. By summarizing and integrating the evidence, 23 best pieces of evidence were formed from 8 aspects: preoperative prevention, posture management, early activity, nutritional intervention, chewing gum, beverage intervention, appropriate traditional Chinese medicine techniques, and health education.

**Conclusion:**

This study summarizes the best evidence for non-pharmacological intervention of postoperative abdominal distension after gynecological laparoscopy from eight aspects. This evidence summary possesses high scientific rigor and clinical applicability, providing guidance for medical staff in constructing standardized postoperative abdominal distension management protocols, thereby facilitating the improvement of patients’ postoperative recovery experience. However, the findings are based on previously published evidence and have not been prospectively validated in clinical practice; therefore, the recommendations should be adapted to local clinical contexts and further evaluated in implementation studies.

**Systematic Review Registration:**

http://ebn.nursing.fudan.edu.cn/home, identifier ES202610098.

## 1 Introduction

With the advancement of minimally invasive techniques, laparoscopic surgery has become a major approach for treating gynecological diseases due to its minimal invasiveness and rapid recovery (1–3). However, postoperative abdominal distension remains one of the most common complications following gynecologic laparoscopy (4). It is associated with multiple factors, including pneumoperitoneum, residual gas, and impaired gastrointestinal motility (5). The incidence can reach 70%–80%, typically occurring within 24–72 h after surgery (6). Postoperative abdominal distension not only causes significant abdominal discomfort and pain but also increases intra-abdominal pressure, affects respiratory function, and delays the recovery of gastrointestinal function, leading to decreased appetite and weakened digestive function. In addition, persistent abdominal distension may also induce negative emotions such as anxiety and irritability. It can increase the risk of postoperative complications such as intestinal obstruction, thereby prolonging hospitalization time and increasing medical burden (7).

Enhanced Recovery After Surgery (ERAS) has been widely applied in gynecologic perioperative care (8–10). Although pharmacological treatments can alleviate symptoms, their potential adverse effects and limited compliance restrict their use (11). In contrast, non-pharmacological interventions, characterized by safety and low cost, have gained increasing attention (12, 13). Studies have shown that interventions such as chewing gum and acupoint stimulation may promote gastrointestinal recovery and reduce abdominal distension (14, 15). However, the current evidence remains fragmented, and standardized management strategies are lacking. Existing ERAS guidelines mainly provide extensive perioperative recommendations, and most systematic reviews focus on a single intervention measure. Although the summary of previous evidence involves postoperative gastrointestinal function management in gynecological laparoscopic patients, postoperative bloating has not yet been identified as a specific clinical issue that requires targeted prevention and management. Therefore, this study aimed to synthesize the currently available published evidence on non-pharmacological interventions for postoperative abdominal distension in gynecologic laparoscopic patients, summarize the reported timing, precautions, and applicability considerations of relevant interventions, and provide an evidence-informed reference for developing context-specific clinical management strategies. This study was registered with the Evidence-Based Nursing Center at Fudan University on March 16, 2026 (registration no. ES202610098).

## 2 Data and methods

### 2.1 Evidence-based question formulation

We used the PIPOST framework to define the evidence question (16): (1) population (P): patients undergoing gynecological laparoscopic surgery; (2) intervention (I): focuses on non-pharmacological strategies for the prevention and management of postoperative abdominal distension, such as early mobilization, nutritional management, chewing gum, and traditional Chinese medicine techniques; (3) professional (P): include healthcare professionals such as doctors and nurses involved in perioperative care; (4) outcomes (O): at the patient level include the incidence of abdominal distension, recovery of gastrointestinal function (e.g., time to first flatus), and length of hospital stay, at the practitioner level, outcomes involve the awareness, acceptance, and implementation of evidence-based practices, while at the system level, outcomes relate to the establishment of standardized perioperative management protocols; (5) setting (S): Gynecology Ward; and (6) type of evidence (T): clinical decision-making tools, guidelines, evidence summaries, systematic reviews, or expert consensus.

### 2.2 Search strategy

According to the 5S evidence resource model (17), evidence retrieval is searched from the top down. The databases searched included: BMJ Best Practice, Up To Date, National Institute of Health and Clinical Excellence (NICE), National Guideline Clearinghouse, Guideline International Network, Scottish Intercollegiate Guidelines Network, Registered Nurses’ Association of Ontario, Chinese Medlive Guideline Network (CMGN), Australian JBI Evidence Based Health Care Database, Cochrane Library, PubMed, CINAHL, Embase, Web of Science, China Biology Medicine, China Knowledge Resource Integrated Database (CNKI), Wanfang and VIP. The search term is constructed by combining medical subject headings (MeSH) with free words. The search formula example is as follows: (“gynecological laparoscopic surgery” or “laparoscopic surgery”) AND (“bloating” or “abdominal bloating” or “gastrointestinal dysfunction” or “postoperative intestinal obstruction” or “delayed defecation” or “disappearance of bowel sounds” or “gastrointestinal diseases”) AND (“non-pharmacological intervention” or “nursing intervention” or “early activity” or “chewing gum” or “acupoint massage” or “dietary management” or “acupoint stimulation” or “traditional Chinese medicine treatment” or “position management” or “ear acupressure” or “exercise therapy”). The search time range is from the database’s establishment to January 31, 2026. The search strategy is provided in the Supplementary material.

### 2.3 Inclusion and exclusion criteria for literature

Inclusion criteria: (1) Patients undergoing gynecological laparoscopic surgery; (2) Studies involving perioperative non-pharmacological interventions; (3) Outcomes related to postoperative abdominal distension or gastrointestinal function recovery; (4) Study types including guidelines, expert consensus, evidence summaries, systematic reviews, and meta-analyses; (5) Publications in English or Chinese.

Exclusion criteria: (1) Studies not relevant to the research topic; (2) Conference abstracts, study protocols, or guideline interpretations; (3) Studies with insufficient data, unavailable full text, or low methodological quality.

All retrieved records were screened independently by two reviewers in a staged process based on the pre-defined inclusion and exclusion criteria. Any disagreements were resolved through consultation with a third reviewer with expertise in evidence-based practice and research methodology. Duplicate records were identified and removed using EndNote software.

### 2.4 Quality assessment of the included studies

#### 2.4.1 Quality assessment procedure

Except for the clinical practice guidelines, which were independently appraised by four researchers trained in evidence-based nursing, all other types of literature were independently evaluated by two trained researchers. If there are disagreements during the evaluation process, a third-party researcher with experience in evidence-based research training will discuss and make the final decision. When there is a discrepancy in conclusions between the extracted evidence, the inclusion of evidence follows the following principles: prioritizing evidence-based evidence, prioritizing high-quality evidence, and prioritizing the latest published and authoritative literature.

#### 2.4.2 Quality assessment tools

Guidelines were appraised using the Appraisal of Guidelines for Research and Evaluation II (AGREE II) instrument (18). The tool consists of 23 items in six areas; each item is evaluated on a scale of 1–7 (1 = strongly disagree, 7 = strongly agree), and the score for each area is a standardized percentage of the sum of the scores for each item in the area. Standardized percentage for each field = (obtained score-least possible score)/(maximum possible score-least possible score) × 100%.

Systematic reviews were critically appraised using the Joanna Briggs Institute (JBI) Critical Appraisal Checklist for Systematic Reviews and Research Syntheses (2017 edition). The checklist comprises 11 items covering the clarity of the review question, appropriateness of the inclusion criteria and search strategy, adequacy of the sources searched, appraisal of included studies, data extraction and synthesis methods, assessment of publication bias, and consistency between the evidence presented and the conclusions drawn. Each item was rated as “Yes,” “No,” “Unclear,” or “Not applicable.” Studies with “No,” “Unclear,” or “Not applicable” ratings were further discussed by the review team to determine whether additional information was required or whether the study should be retained or excluded (19).

Expert consensus statements were assessed using the Joanna Briggs Institute (JBI) expert consensus appraisal criteria (20). This tool contains six evaluation items that can provide judgments of “yes,” “no,” “unclear,” and “not applicable.”

For best practice statements and evidence summaries, the original sources were traced and appraised using appropriate tools according to the study design.

### 2.5 Evidence extraction, synthesis, and grading

Two researchers independently screened the retrieved literature according to predefined inclusion and exclusion criteria. Any disagreements were resolved through discussion or by consulting a third reviewer. Data extraction was then performed independently by the same researchers using standardized forms. Extracted information included study characteristics such as the first author, publication year, source, type of evidence, and study topic. To identify and manage overlapping evidence, the underpinning sources cited in guidelines, evidence summaries, and best-practice documents were traced where available. When multiple documents were based on the same systematic review or overlapping primary studies, they were considered to represent the same body of evidence and were not counted repeatedly during evidence synthesis. When similar recommendations were identified across multiple sources, priority was given to evidence with higher methodological quality, greater direct relevance to gynecologic laparoscopic patients with postoperative abdominal distension, more recent evidence searches or publication dates, and clearer clinical applicability. Where recommendations were inconsistent, the reasons for inconsistency, including differences in populations, interventions, timing, or outcomes, were considered during synthesis. The included evidence was classified and graded according to the Joanna Briggs Institute (JBI) evidence grading system (21). Evidence levels were categorized from Level 1 to Level 5 based on study design, and recommendations were determined using the JBI FAME framework (feasibility, appropriateness, meaningfulness, and effectiveness), resulting in Grade A (strong recommendation) or Grade B (weak recommendation).

## 3 Results

### 3.1 Characteristics of included literature

**FIGURE 1:**
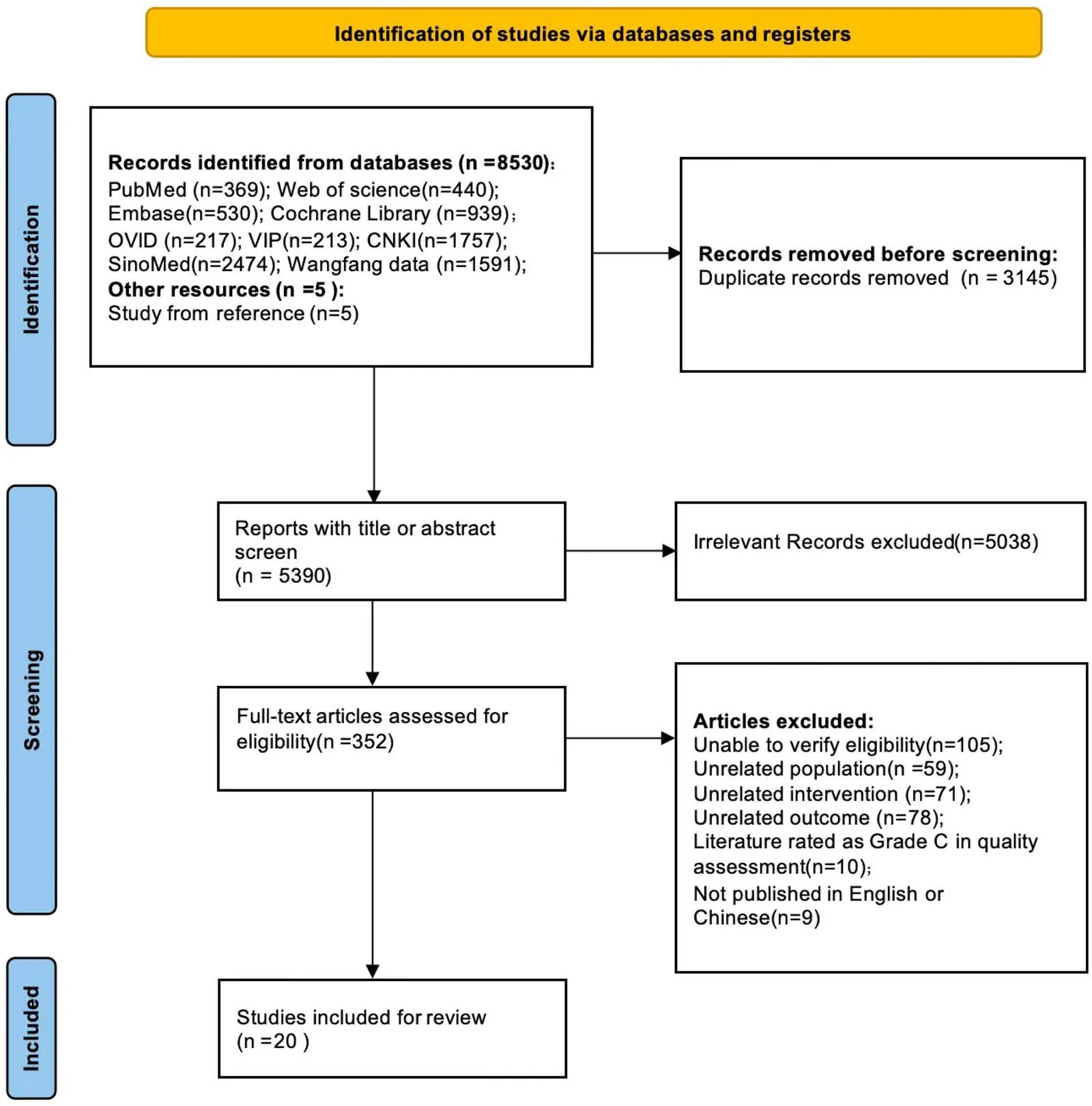
Flow diagram of literature selection.

A total of 8,535 records were identified, of which 3,145 duplicates were removed. After screening 5,390 titles and abstracts, 5,038 records were excluded. Subsequently, 352 full-text articles were assessed for eligibility. Of these, 332 articles were excluded because their eligibility could not be verified (*n* = 105), the population was unrelated to the review question (*n* = 59), the intervention was unrelated (*n* = 71), the outcome was unrelated (*n* = 78), the articles were rated as Grade C in the quality assessment (*n* = 10), or they were not published in English or Chinese (*n* = 9). Finally, 20 studies were included in the review. Finally, 20 studies met the inclusion criteria, including 4 guidelines (9, 10, 22, 23), 5 expert consensus documents (8, 24–27), 1 clinical decision (28), 1 evidence summary (29), and 9 systematic reviews (4, 14, 15, 30–35). The literature screening process is shown in Figure 1, and the overall information for the included literature is shown in Table 1.

**TABLE 1:** Basic characteristics of included literature (*n* = 20).

Included studies	Publication year	Document type	Source	Document topic
Nelson et al. (10)	2023	Guideline	PubMed	ERAS Society Gynecological Oncology Accelerated Rehabilitation Surgery Guidelines: Response to Implementation Challenges -2023 Updated Edition
Stone et al. (9)	2021	Guideline	PubMed	Minimally invasive gynecological surgery enhanced recovery and surgical optimization protocol
Bisch et al. (22)	2018	Guideline	PubMed	Enhanced Recovery Surgery (ERAS) in Gynecological Oncology: System-level Implementation and Audit Promote Medical Value Enhancement and Patient Outcome Improvement
Nelson et al. (23)	2019	Guideline	PubMed	Gynecologic Oncology Perioperative Nursing Guidelines: Enhanced Recovery After Surgery (ERAS) Social Recommendations- 2019 Update
Trabuco and Scheib (28).	2026	Clinical Decision	Up to Date	Elements and Implementation of Accelerated Rehabilitation Program after Gynecological Surgery
Gynecological Pelvic Floor Group, Obstetrics and Gynecology Branch, Chinese Medical Association (24).	2024	Expert consensus	Up to Date	Chinese expert consensus on accelerating recovery through gynecological pelvic floor reconstruction surgery
Santiago et al. (8)	2022	Expert consensus	PubMed	Research on Multidisciplinary Management Path of Gynecological Surgery Perioperative Period Based on ERAS Concept
Chinese and Western Integrated Ovarian Cancer Professional Committee of China Anti-Cancer Association. (25).	2025	Expert consensus	Wanfang	Consensus of Chinese experts on intestinal preparation for gynecological surgery
Chinese Medical Association Obstetrics and Gynecology Branch Accelerated Rehabilitation Surgery Collaboration Group (26).	2019	Expert consensus	Wanfang	Chinese expert consensus on accelerating rehabilitation through gynecological surgery
Bo et al. (27)	2019	Expert consensus	Wanfang	Consensus of Chinese experts on accelerating rehabilitation and gynecological perioperative nursing
Zhou et al. (29)	2023	Evidence summary	Wanfang	Summary of Evidence on Gastrointestinal Function Management during the Perioperative Period of Gynecological Laparoscopy
Tuscharoenporn et al. (14)	2024	Systematic review	Cochrane Library	The effect of chewing gum on gastrointestinal function recovery after laparoscopic gynecological surgery: a systematic review and meta-analysis of randomized controlled trials
Li et al. (30)	2025	Systematic review	PubMed	The impact of nutritional intervention on postoperative prognosis of gynecological cancer patients: systematic review and meta-analysis
O’Neill et al. (31)	2023	Systematic review	PubMed	The impact of accelerated recovery after gynecological surgery: systematic review and meta-analysis
Douligeris et al.(32)	2023	Systematic review	PubMed	The effect of chewing gum on gastrointestinal function recovery after gynecological laparoscopic surgery: a meta-analysis of randomized controlled trials
Wu et al.(33)	2022	Systematic review	PubMed	Meta-analysis of accelerated recovery from gynecological surgery during the perioperative period
Chen et al.(4)	2022	Systematic review	PubMed	The impact of postoperative accelerated recovery plan on postoperative recovery after laparoscopic myomectomy: systematic review and meta-analysis
Salamah et al.(15)	2022	Systematic review	PubMed	The effect of acupoint pressure on postoperative nausea and vomiting in laparoscopic surgery patients: a meta-analysis of randomized controlled trials
Cornwall et al.(34)	2020	Systematic review	PubMed	The effect of coffee on intestinal obstruction after abdominal surgery: a systematic review and meta-analysis of randomized controlled trials
Huang et al.(35)	2021	Systematic review	CNKI	Meta analysis of acupoint application promoting gastrointestinal function recovery after gynecological laparoscopic surgery

### 3.2 Quality appraisal results

#### 3.2.1 Guidelines

Four guidelines were included in the quality appraisal. Their methodological quality was evaluated using the AGREE II instrument, which includes 23 items across six domains and uses a 7-point scoring system. Standardized domain scores were calculated according to the AGREE II manual. Among the four included guidelines, three scored ≥ 60% in all six domains and were classified as Grade A recommendations, indicating relatively high methodological quality (9, 10, 23). One guideline 24 was classified as Grade B (22). Detailed standardized scores for each domain and the overall recommendation grades are shown in Table 2.

**TABLE 2:** Methodological quality evaluation results of the guidelines.

Included literature	Percentage of field standardization %						≥ 60% field number (*n*)	≥ 30% field number (*n*)	Recommendation level
	Scopes and objects	Participant	Rigor of the guideline	Clarity of guidelines	Application of guidelines	Independence of the guide			
Nelson et al.(10)	66.67	75.00	67.86	76.19	67.86	71.43	6	6	A
Stone et al.(9)	76.19	75.00	71.43	66.67	82.14	78.57	6	6	A
Bisch et al.(22)	66.67	83.33	53.32	77.78	44.17	75.00	4	6	B
Nelson et al.(23)	76.19	78.57	75.00	71.43	64.29	71.43	6	6	A

Standardization percentage of each field = (obtained score - minimum possible score)/(maximum possible score - minimum possible score) × 100%; Recommendation level: if the standardized percentage of six fields is > 60%, it is highly recommended (level A); if > 3 areas have a standardized percentage > 30% and < 60% are recommended (level B); if there are ≥ 3 areas with a standardized percentage < 30%, it is not recommended (level C).

#### 3.2.2 Expert consensus statements

Five expert consensus articles were independently evaluated by two evaluators using the JBI expert opinion quality assessment tool, and the overall quality was rated as moderate or high; they were approved for inclusion. Please refer to Table 3 for the detailed quality assessment.

**TABLE 3:** Methodological quality evaluation of expert consensus.

Expert consensus	Evaluation entry						Overall quality
	①	②	③	④	⑤	⑥	
Gynecological Pelvic Floor Group, Obstetrics and Gynecology Branch, Chinese Medical Association (24).	Yes	Yes	Yes	Yes	Yes	Yes	High
Santiago et al.(8)	No	Yes	Yes	Yes	Yes	Yes	Medium
Chinese and Western Integrated Ovarian Cancer Professional Committee of China Anti-Cancer Association (25).	Yes	Yes	Yes	Yes	Yes	Yes	High
Chinese Medical Association Obstetrics and Gynecology Branch Accelerated Rehabilitation Surgery Collaboration Group (26).	Yes	Yes	Yes	Yes	Yes	No	Medium
Bo et al.(27)	Yes	Yes	Yes	Yes	Yes	Yes	High

① Is the source of the opinion clearly stated? ② Are the opinions from influential experts in the field? ③ Are the opinions presented centered on the interests of the people involved in the study? ④ Is the stated conclusion based on the results of the analysis? Are opinions expressed logically? ⑤ Whether to refer to other existing literature? ⑥ Are there any inconsistencies between the opinions presented and the previous literature?

#### 3.2.3 Evaluation of systematic review quality

Nine systematic reviews were assessed using the JBI Critical Appraisal Checklist for Systematic Reviews and Research Syntheses. They focused on postoperative gastrointestinal recovery and abdominal distension and evaluated enhanced recovery after surgery pathways, chewing gum, nutritional interventions, acupoint related therapies, and coffee consumption. Six reviews were rated as having high methodological quality and three as having moderate methodological quality. Detailed results are presented in Table 4.

**TABLE 4:** Methodological quality evaluation results of systematic review.

Systematic review	Evaluation entry											Overall quality
	①	②	③	④	⑤	⑥	⑦	⑧	⑨	⑩	⑪	
Tuscharoenporn et al. (14)	Yes	Yes	Yes	Yes	Yes	Yes	Yes	Yes	Yes	No	Yes	High
Li et al. (30)	Yes	Yes	Yes	Yes	Yes	Yes	Yes	Yes	Yes	No	Yes	High
O’Neill et al. (31)	Yes	Yes	Yes	No	No	Yes	Yes	Yes	Yes	Yes	Yes	Medium
Douligeris et al. (32)	Yes	Yes	Yes	No	Yes	Yes	Yes	Yes	Yes	Yes	Yes	High
Wu et al. (33)	No	Yes	Yes	No	No	Yes	Yes	Yes	Yes	Yes	No	Medium
Chen et al. (4)	Yes	Yes	Yes	Yes	Yes	Yes	Yes	Yes	Yes	No	Yes	High
Salamah et al. (15)	Yes	Yes	Yes	No	Yes	Yes	Yes	Yes	Yes	No	Yes	Medium
Cornwall et al. (34)	Yes	Yes	Yes	Yes	Yes	Yes	Yes	Yes	Yes	Yes	Yes	High
Huang et al. (35)	Yes	Yes	Yes	No	Yes	Yes	Yes	Yes	Yes	No	Yes	High

① Is the review question clearly and explicitly stated? ② Were the inclusion criteria appropriate for the review question? ③ Was the search strategy appropriate? ④ Were the sources and resources used to search for studies adequate? ⑤ Were the criteria for appraising studies appropriate? ⑥ Was critical appraisal conducted by two or more reviewers independently? ⑦ Were there methods to minimize errors in data extraction? ⑧ Were the methods used to combine studies appropriate? ⑨ Was the likelihood of publication bias assessed? ⑩ Were recommendations for policy and/or practice supported by the reported data? ⑪ Were the specific directives for new research appropriate?

#### 3.2.4 Quality appraisal for the clinical decision

One clinical decision was included in this review. The original studies underpinning the clinical decision were traced and appraised using the appropriate JBI critical appraisal tools according to their study designs. The methodological quality of the underpinning evidence was considered high. Therefore, the clinical decision was retained for evidence synthesis.

#### 3.2.5 Quality evaluation results of the evidence summary

One evidence summary was included in this review. The original literature supporting the evidence summary was traced and appraised using the relevant JBI critical appraisal tools according to the study design. The overall methodological quality of the underpinning evidence was high, and the evidence summary was therefore considered eligible for inclusion in the evidence synthesis.

### 3.3 Evidence description and summary

Relevant evidence was extracted from the included studies and appraised using the JBI evidence grading and recommendation system. A total of 23 best evidence statements were synthesized across eight domains: preoperative prevention, posture management, early activity, nutritional interventions, chewing gum, beverage interventions, traditional Chinese medicine techniques, and health education. The JBI evidence hierarchy was used to classify evidence into five levels (21)—Level 1 (randomized controlled trials), Level 2 (quasi-experimental studies), Level 3 (observational studies), Level 4 (descriptive studies), and Level 5 (expert opinion). For clinical guidelines and expert consensus documents, the original cited references were traced, and their respective evidence levels were retained. The details are presented in Table 5.

**TABLE 5:** Evidence summary of non-pharmacological interventions for postoperative abdominal distension following gynecologic laparoscopic surgery.

Evidence items	Evidence content	Level of evidence	Recommended level
Preoperative prevention	1. Avoid routine preoperative preparation and reduce mechanical irritation to the gastrointestinal tract (10).	1	A
	2. For non-diabetic patients without gastrointestinal dysfunction, preoperative fasting should follow current guidelines, with solid foods withheld for at least 6 h ( ≥ 8 h for fatty or fried foods) and clear liquids up to 2 h before anesthesia induction. Additionally, preoperative carbohydrate loading (the night before and 2–3 h before surgery) is recommended to reduce hunger, improve patient comfort, decrease insulin resistance, and promote metabolic stability (8, 25).	5	A
	3. Walk at a speed of 3 km/h for 30 min before surgery to warm up for postoperative gastrointestinal function recovery and reduce postoperative gas accumulation (29).	1	A
	4. Preoperative chewing gum (30–60 min) is recommended to activate gastrointestinal motility through a sham feeding–induced vagal reflex, thereby facilitating postoperative bowel function recovery (31).	1	A
	5. Preoperative auricular acupressure targeting specific acupoints (e.g., sympathetic and Shenmen) is recommended to reduce postoperative gastrointestinal complications and accelerate bowel function recovery; procedures should follow standardized protocols (9, 22).	1	A
	6. Strengthen preoperative health education, inform patients of the causes and preventive measures of postoperative bloating, and combine various forms of education such as pictures and educational videos to enhance patients’ awareness and compliance (29).	1	A
Postoperative management			
Stage position management	7. After returning to the ward, patients are encouraged to adopt a comfortable position (supine or with the head of the bed elevated) and engage in early in-bed activities (26).	5	A
	8. After 6 h postoperatively, when the patient is conscious and their vital signs are stable, it is recommended to adopt a knee chest position for 4–5 min, and then switch to alternating left and right positions to promote anal exhaust and defecation (10).	1	A
Early activities	9. Early mobilization within 24 h after surgery is recommended, beginning with short periods of ambulation and gradually increasing to 4–6 h per day, to promote gastrointestinal function recovery and reduce postoperative complications (8, 25).	5	A
Early nutritional intervention	10. Early enteral nutrition on the day of surgery is recommended for patients without contraindications, such as intestinal obstruction, perforation, severe ileus, or hemodynamic instability, as it is safe and promotes gastrointestinal function recovery (25, 26).	5	B
	11. In patients without nausea or vomiting, oral intake can be initiated with small amounts of clear fluids, followed by a stepwise progression to liquid, semi-liquid, and regular diets within 24 h after surgery. Oral intake should be prioritized, and enteral nutrition supplementation is recommended if energy intake is < 60% of requirements (4, 31).	1	A
Chewing gum	12. After surgery, chewing gum can be started 6 h after anesthesia or on the first day after surgery, for 30 min each time, 3–6 times a day, to promote gastrointestinal function recovery (22, 32).	1	B
	13. Chewing gum, as a form of sham feeding, is recommended to promote postoperative gastrointestinal function and can be used as an adjunct for patients unable to tolerate early oral intake.(8, 24).	5	A
	14. Chewing gum is recommended to reduce postoperative inflammation and the risk of ileus; however, it should be avoided in patients with contraindications (e.g., persistent vomiting, paralytic ileus, oral disorders, or gum allergy), and used with caution to prevent aspiration.(4, 14, 33).	1	A
Beverage intervention	15. Drink caffeinated coffee at 6, 12, and 18 h after surgery (34).	1	A
	16. Postoperative coffee consumption is recommended to promote gastrointestinal function recovery by stimulating colonic motility, particularly in patients who can tolerate oral intake (10, 23).	1	A
	17. It is recommended that patients start drinking 100–150 mL of coffee on the morning of the first day after surgery, three times a day, and finish drinking within 10–20 min (34).	1	A
Appropriate techniques in traditional Chinese medicine	18. Acupoint massage initiated 6 h after surgery is recommended to promote gastrointestinal function recovery; commonly used acupoints include Zusanli, Neiguan, Sanyinjiao, and Zhongwan, with repeated stimulation applied until bowel function returns (15, 35).	1	A
	19. Acupoint application during the early postoperative period is recommended to promote gastrointestinal function recovery; commonly used acupoints include Zusanli, Shangjuxu, and Neiguan, and procedures should follow standardized protocols (28, 31).	1	A
	20. Moxibustion can shorten the first bowel sound and first exhaust time. Commonly used acupoints include Zusanli, Shenque, and Zhongwan acupoints (15, 35).	1	A
	21. Auricular acupressure initiated 6 h after surgery is recommended to promote gastrointestinal recovery; selected acupoints (e.g., stomach, spleen, and sympathetic) may be stimulated repeatedly during the early postoperative period, with monitoring for potential local adverse reactions (15).	1	A
Health education	22. Postoperative health education is recommended as a routine intervention to promote gastrointestinal recovery, including guidance on symptom monitoring, positioning, early mobilization, dietary management, and recognition of abnormal conditions (8).	5	B
	23. Health education should be individualized according to patient characteristics and involve both patients and caregivers to improve adherence and reduce unplanned readmissions (4, 31).	1	B

## 4 Discussion

### 4.1 Non-pharmacological management of postoperative abdominal distension

Postoperative abdominal distension is a common postoperative discomfort after gynecologic laparoscopic surgery and is associated with pneumoperitoneum, residual intra-abdominal gas, impaired gastrointestinal motility, anesthesia, surgical stress, and reduced postoperative activity (3). Although ERAS pathways have been widely applied in gynecologic perioperative care, existing recommendations are often broad, and evidence specifically targeting postoperative abdominal distension remains relatively scattered. In this study, 20 studies were included, comprising guidelines, expert consensus documents, a clinical decision, an evidence summary, and systematic reviews. Through evidence retrieval, quality appraisal, extraction, and synthesis, 23 evidence statements were summarized across eight domains: preoperative prevention, posture management, early activity, nutritional intervention, chewing gum, beverage intervention, appropriate traditional Chinese medicine techniques, and health education. Compared with evidence focusing on a single intervention, this evidence summary emphasizes multidimensional and continuous non-pharmacological management throughout the perioperative period.

The synthesized evidence suggests that these interventions may contribute to postoperative gastrointestinal function recovery by reducing unnecessary gastrointestinal stimulation, promoting early mobilization, stimulating gastrointestinal motility, supporting early nutritional recovery, and improving patient adherence through health education. Therefore, this evidence summary may provide an evidence-informed reference for medical staff to develop individualized and context-specific management strategies. However, because some evidence was derived from broader perioperative gastrointestinal recovery outcomes rather than abdominal distension specifically, clinical application should consider patient characteristics, surgical type, tolerance, local resources, and institutional protocols.

### 4.2 Synthesis of best evidence on non-pharmacological interventions for postoperative abdominal distension

#### 4.2.1 Preoperative preventive management

Preoperative prevention plays an important role in reducing postoperative abdominal distension following gynecologic laparoscopy. As an important component of non-pharmacological interventions, preoperative prevention is of great significance in reducing perioperative stress responses, maintaining gastrointestinal motility stability, and promoting postoperative gastrointestinal function recovery. Evidence suggests (36) that structured preoperative management can help reduce perioperative stress and support gastrointestinal function recovery. Key strategies include avoiding routine mechanical bowel preparation, implementing optimized fasting protocols, and administering preoperative carbohydrate loading 2–3 h before surgery (37, 38). In addition, early activation measures, such as moderate preoperative ambulation, chewing gum 30–60 min before surgery, and ear acupressure within 24 h preoperatively, may help stimulate gastrointestinal motility and reduce postoperative gas retention. Strengthening preoperative education is also essential to improve patient adherence to ERAS principles, thereby facilitating early postoperative mobilization and feeding.

#### 4.2.2 Position and activity

Evidence indicates that posture and activity interventions play a key role in alleviating postoperative abdominal distension and promoting gastrointestinal recovery after gynecologic laparoscopy (39, 40). Early postoperative positioning, including head-of-bed elevation and in-bed mobilization, can be initiated once patients return to the ward. When vital signs are stable and patients regain consciousness, typically around 6 h postoperatively, short periods of knee–chest positioning (4–5 min) combined with lateral repositioning may facilitate the mobilization and expulsion of residual intra-abdominal gas (10, 26, 29).

Early mobilization is also essential (41). Within 24 h after surgery, patients are encouraged to engage in low-intensity activities, such as standing and short-distance walking for 1–2 h, followed by a gradual increase in activity duration to 4–6 h per day as tolerated (8, 25, 29). These interventions may enhance gastrointestinal motility, promote gas clearance, and reduce the risk of postoperative complications, thereby shortening hospital stay. In addition, preoperative ambulation training may further support postoperative gastrointestinal recovery, suggesting that posture and activity management should be integrated throughout the perioperative period. Individualized and staged intervention strategies should be implemented according to patients’ surgical type, tolerance, and recovery status (42).

#### 4.2.3 Nutritional intervention

Early postoperative nutritional management is essential for patients undergoing gynecologic laparoscopic surgery, provided that their clinical condition is stable and gastrointestinal function has recovered (43, 44). Evidence suggests that, in the absence of contraindications such as nausea, vomiting, or ileus, patients may initiate oral intake with small amounts of warm water (10–20 mL) as early as 2 h after surgery to assess tolerance (4, 31). If well tolerated, a gradual progression to liquid or semi-liquid diets can be introduced within 4–6 h postoperatively, followed by a stepwise transition to soft and regular diets within 24 h according to individual tolerance. For patients with inadequate oral intake or nutritional risk, individualized nutritional support, including oral nutritional supplements or enteral nutrition, should be considered (25, 26, 30). Early nutritional intervention may promote gastrointestinal motility, facilitate gas and intestinal content clearance, and reduce postoperative abdominal distension. In addition, gradual dietary advancement helps avoid gastrointestinal overload, thereby supporting overall recovery.

#### 4.2.4 Chewing gum and beverage intervention

Evidence suggests that chewing gum and beverage interventions are simple, low-cost strategies that can promote postoperative gastrointestinal recovery following gynecologic laparoscopy (45, 46). These interventions may stimulate suppressed gastrointestinal neuroendocrine pathways and facilitate the transition from postoperative ileus to functional recovery. Postoperative abdominal distension is not only related to residual pneumoperitoneum, but also to anesthesia effects, surgical stress, and reduced intestinal motility (29). Therefore, such interventions may contribute to early restoration of gastrointestinal function through mild physiological stimulation. Chewing gum, as a form of sham feeding, can be initiated once patients are awake and clinically stable, typically 6 h to 1 day after surgery, for 30 min per session, 3–6 times daily, until the return of bowel function (32). It may activate vagal reflexes, promote gastrointestinal hormone secretion, and enhance intestinal motility, thereby alleviating abdominal distension and discomfort. Similarly, coffee consumption may be considered for patients who tolerate oral intake (10, 23, 29, 34). Caffeinated coffee can be administered at 6, 12, and 18 h postoperatively, or from the morning of postoperative day 1 (100–150 mL per dose, three times daily). Moderate coffee intake may stimulate gastrin release, enhance colonic motility, and promote bowel movement, thereby reducing abdominal distension.

Overall, chewing gum and beverage interventions may facilitate gastrointestinal recovery through multiple mechanisms and can serve as adjunctive strategies before the resumption of a normal diet. Future studies may further integrate these approaches into standardized ERAS pathways and optimize their application based on individual tolerance and clinical conditions.

#### 4.2.5 Traditional Chinese medicine interventions

Traditional Chinese medicine (TCM) interventions may serve as effective adjunctive strategies for promoting gastrointestinal recovery and alleviating postoperative abdominal distension following gynecologic laparoscopy, particularly during the early postoperative period (47, 48). Evidence suggests that, compared with preoperative prevention, the early postoperative phase is both the peak period for gastrointestinal dysfunction and the optimal window for non-pharmacological intervention. TCM therapies, including acupoint stimulation, ear acupressure, and external applications, may provide gentle and sustained regulation of gastrointestinal function, thereby improving symptoms such as abdominal distension, nausea, and vomiting (15, 35).

Among these, acupoint massage and ear acupressure are commonly applied as core interventions. These techniques can be initiated as early as 6 h postoperatively and may promote gastrointestinal motility, improve local circulation, and regulate gastrointestinal hormone secretion. Acupoint massage typically involves stimulation of commonly used acupoints (e.g., Zusanli, Neiguan, Sanyinjiao, and Zhongwan), with each acupoint pressed for 1–2 min, three times daily, until the return of flatus or bowel function (15, 29, 35). Ear acupressure provides continuous stimulation through auricular points. During the first three postoperative days, pressure may be applied three times daily for 1–2 min per acupoint, alternating between both ears, with local soreness, numbness, distension, pain, or warmth considered appropriate stimulation responses (21). Both approaches are simple, well tolerated, and particularly suitable for patients with limited mobility or delayed oral intake. Additional modalities, such as acupoint application and warming herbal sachets, may be used as complementary interventions. Acupoint application may be performed from postoperative day 1 to day 3, commonly on bilateral Zusanli, Shangjuxu, and Neiguan, once daily for 30 min each time. Warming herbal sachets may be placed near the patient’s nose, every 10 min, lasting 1–2 min per session, while patients are encouraged to take deep breaths to promote qi circulation, strengthen the spleen, and relieve gastrointestinal discomfort (29). These methods may further enhance gastrointestinal motility and modulate inflammatory responses, potentially providing synergistic benefits. Overall, TCM interventions should be considered supportive rather than substitutive therapies and can be integrated into multimodal perioperative management to facilitate early gastrointestinal recovery. Future research may further optimize their application based on individual patient characteristics and clinical conditions.

#### 4.2.6 Health education

Health education is an important component in the management of postoperative abdominal distension following gynecologic laparoscopy. It aims to improve patients’ understanding of the condition and enhance adherence to non-pharmacological interventions. Staged and individualized education, focusing on key aspects such as posture, early mobilization, and dietary progression, may support recovery while enabling patients to recognize warning symptoms (4, 8, 10, 31). Overall, continuous health education may improve compliance, promote gastrointestinal recovery, and reduce postoperative abdominal distension.

## 5 Limitations

This study provides a comprehensive framework of non-pharmacological strategies for postoperative abdominal distension, integrating preoperative prevention, postoperative management, and health education, and may enhance the translational value of the currently available evidence. However, as this evidence summary is based on previously published literature and expert consensus, heterogeneity among studies and the lack of prospective clinical validation may limit the generalizability and clinical applicability of the findings. Future high-quality prospective implementation studies are needed to further validate these interventions in clinical practice.

## 6 Conclusion

This study systematically summarized the currently available published evidence on non-pharmacological interventions for postoperative abdominal distension after gynecological laparoscopic surgery across eight dimensions: preoperative prevention, posture management, early activity, nutritional intervention, chewing gum, beverage intervention, appropriate traditional Chinese medicine techniques, and health education. The synthesized evidence suggests that multidimensional and continuous non-pharmacological interventions during the perioperative period may help promote gastrointestinal function recovery and improve patients’ postoperative recovery experience. In clinical practice, it is recommended that medical staff select and personalize relevant evidence based on local medical conditions, patient characteristics, and individual needs to improve the effectiveness of interventions and nursing quality.

## Data Availability

The original contributions presented in this study are included in this article/Supplementary material, further inquiries can be directed to the corresponding author.

## References

[1] Sheng Y Hong Z Wang J Mao B Wu Z Gou Y et al. Efficacy and safety of robot-assisted laparoscopic myomectomy versus laparoscopic myomectomy: a systematic evaluation and meta-analysis. *World J Surg Oncol.* (2023) 21:230. 10.1186/s12957-023-03104-8 37507735 PMC10375654

[2] Chene G Miquel L Agostini A Bendifallah S Solignac C Darne B et al. Safety of in-bag morcellation during laparoscopic myomectomy and hysterectomy: a systematic review and meta-analysis. *J Minimally Invasive Gynecol.* (2026) 33:164–77. 10.1016/j.jmig.2025.07.002 40623664

[3] Benton-Bryant C Pour N Baekelandt J Elhindi J Ekanyake K Kapurubandara S. Transvaginal natural orifice transluminal endoscopic surgery (vNOTES) in benign gynaecology: a systematic review of adnexal, myomectomy and prolapse procedures. *J Minimally Invasive Gynecol.* (2025) 32:318–51.e2. 10.1016/j.jmig.2024.11.004 39647776

[4] Chen Y Fu M Huang G Chen J. Effect of the enhanced recovery after surgery protocol on recovery after laparoscopic myomectomy: a systematic review and meta-analysis. *Gland Surg.* (2022) 11:837–46. 10.21037/gs-22-168 35694088 PMC9177272

[5] Khalil M Jayne D Chapman S. Decoding postoperative ileus. *Br J Surg.* (2025) 112:znaf237. 10.1093/bjs/znaf237 41248929

[6] Wang B Hu L Hu X Han D Wu J. Exploring perioperative risk factors for poor recovery of postoperative gastrointestinal function following gynecological surgery: a retrospective cohort study. *Heliyon.* (2023) 10:e23706. 2023.e23706 10.1016/j.heliyon.2023.e23706 38205292 PMC10776945

[7] Kirkpatrick A Roberts D De Waele J Jaeschke R Malbrain M De Keulenaer B et al. Intra-abdominal hypertension and the abdominal compartment syndrome: updated consensus definitions and clinical practice guidelines from the world society of the abdominal compartment syndrome. *Intensive Care Med.* (2013) 39:1191–206. 10.1007/s00134-013-2906-z 23673399 PMC3680657

[8] Santiago A Filho AL Cândido E Ribeiro P Silva J Primo W et al. Perioperative management in gynecological surgery based on the ERAS program: number 2 - february 2022. *Rev Bras Ginecol Obstetr.* (2022) 44:202–10. 10.1055/s-0042-1743401 35213920 PMC9948094

[9] Stone R Carey E Fader A Fitzgerald J Hammons L Nensi A et al. Enhanced recovery and surgical optimization protocol for minimally invasive gynecologic surgery: an AAGL white paper. *J Minimally Invasive Gynecol.* (2021) 28:179–203. 10.1016/j.jmig.2020.08.006 32827721

[10] Nelson G Fotopoulou C Taylor J Glaser G Bakkum-Gamez J Meyer L et al. Enhanced recovery after surgery (ERAS§) society guidelines for gynecologic oncology: addressing implementation challenges - 2023 update. *Gynecol Oncol.* (2023) 173:58–67. 10.1016/j.ygyno.2023.04.009 37086524

[11] Hedrick T McEvoy M Mythen MM Bergamaschi R Gupta R Holubar S et al. American society for enhanced recovery and perioperative quality initiative joint consensus statement on postoperative gastrointestinal dysfunction within an enhanced recovery pathway for elective colorectal surgery. *Anesthesia Analgesia*. (2018) 126:1896–907. 10.1213/ANE.0000000000003781 29293183

[12] Arslan H Çelik SŞ Bozkul G. Postoperative ileus and nonpharmacological nursing interventions for colorectal surgery: a systematic review. *J Perianesth Nurs*. (2025) 40:181–94. 10.1016/j.jopan.2024.03.012 38970591

[13] Matsuo K Rau A Ciesielski K Vallejo A Mandelbaum R Roman L et al. Concurrent minimally invasive gynecologic procedures at the time of laparoscopic cholecystectomy. *Obstetr Gynecol.* (2023) 142:1491–5. 10.1097/AOG.0000000000005420 37883996

[14] Tuscharoenporn T Uruwankul K Charoenkwan K. Effects of postoperative gum chewing on recovery of gastrointestinal function following laparoscopic gynecologic surgery: systematic review and meta-analysis of prospective studies. *J Clin Med.* (2024) 13:2851. 10.3390/jcm13102851 38792393 PMC11121968

[15] Salamah H Elsayed E Brakat A Abualkhair K Hussein M Saber S et al. The effects of acupressure on postoperative nausea and vomiting among patients undergoing laparoscopic surgery: a meta-analysis of randomized controlled trials. *Explore*. (2023) 19:301–9. 10.1016/j.explore.2022.10.015 36319586

[16] Zhu Z Hu Y Zhou Y Gu Y Xing W Chen Y et al. Promoting the transformation of evidence to clinical practice: research topic selection and problem construction. *Nurses’ Continuing Educ J.* (2020) 35:796–9. 10.16821/j.cnki.hsjx.2020.09.008

[17] Alper B Haynes RB. EBHC pyramid 5.0 for accessing preappraised evidence and guidance. *Evid Based Med.* (2016) 21:123–5. 10.1136/ebmed-2016-110447 27325531

[18] Brouwers M Kho M Browman G Burgers J Cluzeau F Feder G et al. AGREE II: advancing guideline development, reporting and evaluation in health care. *CMAJ.* (2010) 182:E839–42. 10.1503/cmaj.090449 20603348 PMC3001530

[19] Hilton M. JBI critical appraisal checklist for systematic reviews and research syntheses. *J Can Health Libr Assoc.* (2024) 45:180–3. 10.29173/jchla29801

[20] Yu L Gu W Lu B Xu M Feng Y Men G et al. Summary of the best evidence on parenting stress management for parents of very low birth weight premature infants after discharge. *Nurs Res.* (2026):1519–26.

[21] Yi L Chen Y Hu R. A summary of the best evidence for wet pack management. *Risk Manag Healthc Policy.* (2025) 18:43–52. 10.2147/RMHP.S497773 39802342 PMC11724621

[22] Bisch S Wells T Gramlich L Faris P Wang X Tran D et al. Enhanced recovery after surgery (ERAS) in gynecologic oncology: system-wide implementation and audit leads to improved value and patient outcomes. *Gynecol Oncol.* (2018) 151:117–23. 10.1016/j.ygyno.2018.08.007 30100053

[23] Nelson G Bakkum-Gamez J Kalogera E Glaser G Altman A Meyer L et al. Guidelines for perioperative care in gynecologic/oncology: enhanced recovery after surgery (ERAS) society recommendations—2019 update. *Int J Gynecol Cancer.* (2019) 29:651–68. 10.1136/ijgc-2019-000356 30877144

[24] Urogynecology Subgroup, Chinese Society of Obstetrics and Gynecology, Chinese Medical Association. Chinese expert consensus on enhanced recovery after surgery for pelvic floor reconstructive surgery. *Chinese J Obstetr Gynecol.* (2024) 59:829–38. 39581767 10.3760/cma.j.cn112141-20240610-00323

[25] Zhang Y Lou G Zhang S. Chinese expert consensus on bowel preparation for gynecological surgery (2025 edition). *J Pract Oncol.* (2025) 39:363–71.

[26] Collaborative Group of Accelerated Rehabilitation Surgery, Obstetrics and Gynecology Branch, Chinese Medical Association. Chinese expert consensus on accelerated recovery after gynecological surgery. *Chinese J Obstetr Gynecol.* (2019) 54:73–9. 10.3760/cma.j.issn.0529-567x.2019.02.001 30803164

[27] Bo H Ge L Liu X Zhang M Li Y et al. Expert consensus on perioperative nursing for accelerated recovery in gynecology. *Chinese J Modern Nurs.* (2019) 25:661–8.

[28] UpToDate. *Elements and Implementation of Accelerated Rehabilitation Program after Gynecological Surgery - UpToDate[EB/OL].* (2026). Available online at: http://61.153.6.101:11111/contents/zh-Hans/enhanced-recovery-after-gynecologic-surgery-components-and-implementation?search=%E5%8A%A0%E9%80%9F%E5%BA%B7%E5%A4%8D&source=search_result&selectedTitle=3~150&usage_type=default&display_rank=3 (accessed March 9, 2026).

[29] Zhou M Lu J Ning L Liu S Yu M. Summary of the best evidence for perioperative gastrointestinal function management in patients undergoing laparoscopic surgery for benign gynecological diseases. *Chinese J Modern Nurs.* (2023) 29:4099–107. 10.3760/cma.j.cn115682-20230202-00326

[30] Li W Wang F Guo X Wang Q Wang Y Li R et al. The impact of nutritional intervention on postoperative prognosis in gynecological cancer patients: a systematic review and meta-analysis. *Supportive Care Cancer.* (2025) 33:983–1003. 10.1007/s00520-025-10065-z 41144033

[31] O’Neill A Calpin G Norris L Beirne J. The impact of enhanced recovery after gynaecological surgery: a systematic review and meta-analysis. *Gynecol Oncol.* (2023) 168:8–16. 10.1016/j.ygyno.2022.10.019 36356373

[32] Douligeris A Diakosavvas M Kathopoulis N Kypriotis K Mortaki A Angelou K et al. The effect of postoperative gum chewing on gastrointestinal function following laparoscopic gynecological surgery. A meta-analysis of randomized controlled trials. *J Minimally Invasive Gynecol.* (2023) 30:783–96. 10.1016/j.jmig.2023.06.015 37422054

[33] Wu X Liu L Zhou F. Meta-analysis for the evaluation of perioperative enhanced recovery after gynaecological surgery. *Ginekologia Polska.* (2022) 93:896–903. 10.5603/GP.a2022.0064 36621969

[34] Cornwall H Edwards B Curran J Boyce S. Coffee to go? The effect of coffee on resolution of ileus following abdominal surgery: a systematic review and meta-analysis of randomised controlled trials. *Clin Nutr.* (2020) 39:1385–94. 10.1016/j.clnu.2019.06.003 31253438

[35] Huang Y Wu M Zhao X Chen H Cao L et al. Meta-analysis of acupoint application promoting gastrointestinal function recovery after gynecological laparoscopic surgery. *World Traditional Chinese Med.* (2021) 16:3523–31. 10.3969/j.issn.1673-7202.2021.23.020

[36] Ljungqvist O. Managing surgical stress: principles of enhanced recovery and effect on outcomes. *Clin Nutr ESPEN.* (2025) 67:56–61. 10.1016/j.clnesp.2025.02.023 40058494

[37] Rollins K Javanmard-Emamghissi H Acheson A Lobo D. The role of oral antibiotic preparation in elective colorectal surgery: a meta-analysis. *Ann Surg.* (2019) 270:43–58. 10.1097/SLA.0000000000003145 30570543 PMC6570620

[38] Developed by the Joint Writing Group of the International Urogynecological Association and the American Urogynecologic Society. AUGS-IUGA joint clinical consensus statement on enhanced recovery after urogynecology surgery. *Int Urogynecol J.* (2022) 33:2921–40. 10.1007/s00192-022-05223-4 36161507

[39] Zhou M Fu A Zhang S Ning L. Management of gastrointestinal function in patients after laparoscopic surgery for benign gynaecological diseases: a best practice implementation project. *Nurs Open.* (2026) 13:e70444. 10.1002/nop2.70444 41906267 PMC13140364

[40] Wang B Han D Hu X Chen J Liu Y Wu J. Perioperative liberal drinking management promotes postoperative gastrointestinal function recovery after gynecological laparoscopic surgery: a randomized controlled trial. *J Clin Anesthesia.* (2024) 97:111539. 10.1016/j.jclinane.2024.111539 38945059

[41] Sarkies M Testa L Carrigan A Roberts N Gray R Sherrington C et al. Perioperative interventions to improve early mobilisation and physical function after hip fracture: a systematic review and meta-analysis. *Age Ageing.* (2023) 52:afad154. 10.1093/ageing/afad154 37596922 PMC10439513

[42] Wang J Xu Z Chen F Wei X Shen B Deng X. Advancing perioperative care: introducing patient-centered comfort management. *Gland Surg.* (2025) 14:1391–8. 10.21037/gs-2025-79 40771388 PMC12322764

[43] Nuermanguli R Jing D JiangYing Y Yu H. Application of enhanced recovery after surgery in perioperative management of patients undergoing laparoscopic surgery for benign gynecological conditions. *Medicine.* (2025) 104:e43161. 10.1097/MD.0000000000043161 40696632 PMC12282710

[44] Schneider S Armbrust R Spies C du Bois A Sehouli J. Prehabilitation programs and ERAS protocols in gynecological oncology: a comprehensive review. *Arch Gynecol Obstetr.* (2020) 301:315–26. 10.1007/s00404-019-05321-7 31616986

[45] Kadirogullari P Seckin K Yalcin Bahat P Aytufan Z. The effect of chewing gum on bowel function postoperatively in patients with total laparoscopic hysterectomy: a randomised controlled trial. *J Obstet Gynaecol.* (2022) 42:1192–7. 10.1080/01443615.2025.2484495 34379539

[46] Desgranges F Chassard D Bouvet L. Pre-operative gum chewing: forbidden, allowed or recommended. *Anaesthesia.* (2019) 74:539. 10.1111/anae.14616 30847912

[47] Luo Y Feng X Wu D Wang J Lyv Z Zheng J et al. A randomized controlled trial of chinese traditional medicine dachengqi decoction in the treatment of postoperative intestinal function recovery. *Transl Cancer Res.* (2020) 9:4498–506. 10.21037/tcr-19-2671 35117815 PMC8797330

[48] Liu Y Jin W Wang K Chang Z Zhang L. Traditional chinese medicine nursing for gastrointestinal function recovery in patients with uterine fibroids after high-intensity focused ultrasound treatment. *J Multidisciplinary Healthc.* (2024) 17:6099–108. 10.2147/JMDH.S496714 39734795 PMC11675286

