# Supplementary material for: Best evidence summary of non-pharmacological interventions for postoperative abdominal distension in gynecological laparoscopic
Source: Front Med (Lausanne). 2026 Aug 18;13:1863641. doi: 10.3389/fmed.2026.1863641 (PMC13529959; doi:10.3389/fmed.2026.1863641)
Supplement: Supplementary file 1 [file Table_1.docx]

Supplementary Material

# 1 Search strategies

**Database:** PubMed

**Search date:** 2026.02.05

**Search strategy:** (("Laparoscopes"[Mesh] OR "Gynecology"[Mesh] OR "Endometriosis"[Mesh] OR "Leiomyoma"[Mesh] OR "Hysterectomy"[Mesh] OR "Uterine Cervical Neoplasms"[Mesh] OR "Ovarian Neoplasms"[Mesh] OR Laparoscopes[Title/Abstract] OR pelvic abscess[Title/Abstract] OR Gynecological Laparoscopic Surgical Procedures[Title/Abstract] OR Gynecological laparoscopy[Title/Abstract] OR Gynecology[Title/Abstract] OR Endometrial Cance*[Title/Abstract] OR Hysterectom*[Title/Abstract] OR Ectopic Pregnanci*[Title/Abstract] OR Ectopic Pregnancy[Title/Abstract] OR Cervical Cancer[Title/Abstract] OR Ovarian Cance*[Title/Abstract]) AND ("Complementary Therapies"[Mesh] OR "Aromatherapy"[Mesh] OR "Electric Stimulation Therapy"[Mesh] OR "Acupuncture"[Mesh] OR "Acupuncture Points"[Mesh] OR "Massage"[Mesh] OR "Exercise Therapy"[Mesh] OR "Knee-Chest Position"[Mesh] OR "Hyperthermia, Induced"[Mesh] OR "Moxibustion"[Mesh] OR non-pharmacological[Title/Abstract] OR complementary[Title/Abstract] OR electroacupuncture[Title/Abstract] OR "early feeding"[Title/Abstract] OR "relaxation"[Title/Abstract] OR yoga[Title/Abstract] OR aromatherapy[Title/Abstract] OR "early mobilization"[Title/Abstract] OR alternative[Title/Abstract] OR integrative[Title/Abstract] OR "gum chewing"[Title/Abstract] OR Acupuncture Poin*[Title/Abstract] OR Acupoin*[Title/Abstract] OR Chewing Gu*[Title/Abstract] OR Exercise Therap*[Title/Abstract] OR Rehabilitation Exercis*[Title/Abstract] OR Knee Chest Positio*[Title/Abstract] OR "TCM"[Title/Abstract] OR abdominal massage[Title/Abstract] OR Dietary guidance[Title/Abstract] OR enhanced recovery After Surgery[Title/Abstract] OR "ERAS"[Title/Abstract])) AND ("Flatulence"[Mesh] OR "Defecation"[Mesh] OR "Signs and Symptoms, Digestive"[Mesh] OR "Intestinal Obstruction"[Mesh] OR "Postoperative Nausea and Vomiting"[Mesh] OR Defecatio*[Title/Abstract] OR Bowel Functio*[Title/Abstract] OR Intestinal Obstructio*[Title/Abstract] OR Gastrointestinal function[Title/Abstract] OR Anal exhaust[Title/Abstract] OR Gastrointestinal reactions[Title/Abstract] OR abdominal distension[Title/Abstract] OR Postoperative Ileus[Title/Abstract] OR ( nausea AND vomiting[Title/Abstract]))

**Other English databases:** Search strategies for other English databases (Cochrane Central Register of Controlled Trials, Web of Science, Embase, OVID) were adapted using similar keywords and search logic.

**Database:** Wanfang Database

**Search date:** 2026.02.05

**Search strategy:** （妇科腹腔镜 OR 妇科腹腔镜手术患者 OR 妇科腹腔镜手术 OR卵巢囊肿 OR 子宫内膜异位症 OR 子宫肌瘤 OR 子宫内膜癌 OR 子宫切除术 OR 异位妊娠 OR 卵巢癌 OR 宫颈癌）AND（ 非药物 OR 护理干预 OR 中医适宜技术 OR 穴位 OR 按压 OR 艾灸 OR 耳穴 OR推拿 OR 腹部按摩 OR 腹部热敷 OR 电针 OR 穴位电刺激 OR 咀嚼口香糖 OR 饮食指导 OR 咖啡 OR 早期进水进食物 OR 芳香疗法 OR 音乐疗法 OR 体位改变 OR 早期活动 OR 下床活动 OR 翻身活动 OR 快速康复 OR 加速康复 OR 运动疗法）AND（腹胀 OR 胃肠胀气 OR 腹部胀气 OR 排便推迟 OR 胃肠道反应 OR 腹部膨隆 OR 术后排气延迟 OR 胃肠功能 OR 消化道症状 OR 肠鸣音消失 OR 肠鸣音减弱 OR 肠麻痹 OR 肛门排气 OR 气体释放 OR 肠梗阻）

**Other Chinese databases:** Search strategies for other Chinese databases (SinoMed, China National Knowledge Infrastructure (CNKI)) were adapted using similar keywords and search logic.
